# *Zataria multiflora* Boiss. Essential Oil Induce Apoptosis in Two Human Colon Cancer Cell Lines (HCT116 & SW48)

**Published:** 2020-04

**Authors:** Narges AHANI, Mohammad Hossein SANGTARASH, Majid ALIPOUR ESKANDANI, Massoud HOUSHMAND

**Affiliations:** 1. Department of Biology, Faculty of Sciences, University of Sistan and Baluchestan, Zahedan, Iran; 2. Department of Food Hygiene and Quality Control, Faculty of Veterinary Medicine, University of Zabol, Zabol, Iran; 3. Department of Medical Genetics, National Institute of Genetic Engineering and Biotechnology, Tehran, Iran; 4. Research Center, Knowledge University, Erbil, Kurdistan Region, Iraq

**Keywords:** *Zataria multiflora* Boiss., Essential oil, Apoptosis, Colon cancer

## Abstract

**Background:**

*Zataria multiflora* Boiss. is known by the common Persian name “*Avishan-e-Shirazi*”, is one of the best-known medicinal herbs belonging to the Labiatae (Lamiaceae) family. The aim of this study was to evaluate the anticancer effects and the underlying mechanisms of how *Z. multiflora* Boiss., essential oil induced apoptosis in the human colorectal tumor cell lines (HCT116 & SW48).

**Methods:**

This study was conducted in National Institute of Genetic Engineering and Biotechnology (NIGEB) (Tehran, Iran) from 2017 to 2019. The cytotoxicity of this essential oil was assessed by 3- (4,5-di-methylthiazol-2-yl)- 2,5-diphenyltetra-zolium bromide *(*MTT) assay, trypan blue exclusion, and colony formation assays. We assessed apoptosis and measure intracellular reactive oxygen species (ROS) by flow cytometry. Then gene expression was analyzed by Quantitative Real-Time RT-PCR.

**Results:**

*Z. multiflora* Boiss., essential oil time- and dose-dependently inhibits cell proliferation and also induced apoptosis in both cell lines via UCP_2_-related mitochondrial pathway by the induction of intracellular ROS.

**Conclusion:**

*Z. multiflora* Boiss., essential oil could be a good candidate for use as an inhibitor of the growth of colorectal tumor cells.

## Introduction

Colorectal cancer (CRC) is the fourth leading cause of cancer death in women, behind breast, lung, and cervical cancer, respectively and second leading cause of cancer death in men, behind only lung cancer, in the world (1). There are different approaches to treat colorectal cancer, based on the type and stage of colorectal cancer, including surgery, radiation therapy, chemotherapy, targeted therapy, immunotherapy etc. (1).

Natural compounds derived from plants have been regarded as potential chemopreventive and chemotherapeutic compounds in the last decade (2). Due to they are relatively less toxic to healthy cells, and they are cost-effective and better accepted by patients, they are now playing an attractive role for the development of tumor treatment and prevention strategies (3, 4). *Zataria multiflora’*s English name is Wild marjoram and its botanical name is *Zataria multiflora* Boiss and is known by the common Persian name “*Avishan-e-Shirazi*”. *Z. multiflora* Boiss., is one of the best-known medicinal herbs belonging to the Labiatae (Lamiaceae) family widely distributed in Iran, Pakistan and Afghanistan (5). This plant is not only a popular food flavor ingredients but is also well known for its diverse beneficial effects, including pharmaceutical, antimicrobial, anti-nociceptive, antioxidant and anti-inflammatory properties (6). The biological properties of Z. *multiflora* have been attributed to the essential oil that has phenolic compounds such as carvacrol and thymol. *Z. multiflora* essential oil is extracted from the flowered branches of the plant (5, 6). This essential oil is a growth inhibitor that currently bears clinical assessment for several malignancies such as cervical cancer, breast cancer (7, 8). However, the effect of this essential oil still needs to be elucidated in colorectal cancers.

UCP_2_ belongs to a family of anion carrier proteins located in the inner mitochondrial membrane (9). UCP2 is over-expressed in various types of cancer. UCP_2_ negatively regulates the production of reactive oxygen species (ROS) in the mitochondrion (10, 11). The BCL-2 family members can be divided into three groups: anti-apoptotic proteins (such as *BCL-2* (B-cell lymphoma 2), *BCL-XL* (B-cell lymphoma-extra-large)), pro-apoptotic effectors (such as *Bax* (BCL2 associated X protein), *Bak* (BCL2 antagonist/killer)), and pro-apoptotic activators (such as *Bad* (Bcl2-associated agonist of cell death), ***Bik* (**BCL2 interacting killer)) (11). Therefore, we assessed the expression of *Ucp2, Bcl2, Bax, Bak, bik, Cytochrome c* genes.

The present study aimed to investigate the cytotoxic effects and also the underlying mechanisms of how *Z. multiflora* Boiss., essential oil induced apoptosis in human colorectal cancer cell lines (HCT116 & SW48).

## Materials & Methods

### Cell lines and Monolayer culture

HCT116 and SW48 human colorectal cancer cell lines were purchased from the National Cell Bank, Pasteur Institute of Iran (Tehran, Iran). HCT116 cell line was cultured in Dulbecco’s modified Eagle’s medium (DMEM F-12 + Glutamax) (Bio-idea, Iran) (Cat No. BI 1027) and SW48 Cell line was cultured in Roswell Park Memorial Institute medium (RPMI 1640+Glutamax) (Bio-idea, Iran) (Cat No. BI 1031), they were supplemented with 10% fetal bovine serum (FBS) (Gibco) (Cat No. 10270-106) and Pen Strep (Penicillin Streptomycin) (100x) (10,000 Units/ml Penicillin, 10,000 μg/ml Streptomycin) (Bio-idea, Iran) (Cat No. BI 1036).

### Treatment of cells with *Z. multiflora Boiss.* essential oil

*Z. multiflora Boiss.,* essential oil was purchased from Barij Essence Pharmaceutical Company, Kashan, Iran. Next, serial dilutions of this essential oil were prepared in culture medium, DMEM and RPMI 1640, to get the desired concentrations ranged from 2.5 to 250 ppm.

### Microculture Tetrazolium (MTT) Assay

To determine cell viability *3- (4,5-di-methylthiazol-2-yl)- 2,5-diphenyltetra-zolium bromide (MTT) assay* was used. HCT116 and SW48 cell lines (8000 cells/well) were seeded in 96-well plates (SPL Life Sciences, Pocheon, Korea) and incubated in a CO_2_ incubator at 37 °C. After 24 h the original medium was removed and 100 μl fresh medium was added, then cells were treated with *Z. multiflora* Boiss. essential oil (with concentrations of 0, 2.5, 5, 10, 20, 30, 40, 50, 100, 150, 200, 250 ppm) and incubated for 24, 48 and 72 h, after this time the medium was replaced with ready-To-Use RPMI 1640. Then 10 μl of the 12mM MTT solution (Bio-idea, Iran) (Cat No. BI1017) was added to each well and the plates were incubated for 4 h. After that, medium was removed from the wells, and then 50 μl of Dimethyl sulfoxide (DMSO) solution was added to each well, to solve precipitated formazan. Then plates were incubated for 10 min. Finally, the optical densitometry was measured by ELISA reader at a wavelength of 570 nm. The percentage of cell viability was calculated as (%)=(optical density of treated cells/optical density of untreated cells) × 100. The survival curves of HCT116 and SW48 cell lines were constructed based on different concentrations of extract after the specified period. The effects of essential oil were determined by IC_50_ values (the concentration of essential oil that reducing the absorbance of treated cells by 50% with respect to untreated cells) (12, 13).

### Trypan Blue Exclusion Test

To discriminate between viable and non-viable cells, trypan blue exclusion test was used. Approximately 1 × 10^5^ of HCT116 & SW48 cells were seeded per well in 6-well plates and incubated. After 24 h, the cells were treated with *Z. multiflora* Boiss., essential oil (with concentrations of 0, 20, 40, 50, 100, 250 ppm) and incubated. Overall, 48 h after treatment, Cells were harvested by digestion with 0.25% trypsin-EDTA solution (1X) (Bio-idea, Iran) (Cat No. BI1002). Then, harvested cells were resuspended in phosphate-buffered saline (PBS) (Bio-idea, Iran) (Cat No. BI-1038-500), and then 10 μl of cell suspension were mixed with 10 μl of 0.4% trypan blue dye (Bio-idea, Iran) (Cat No. BI1014), then they were mixed and incubated at room temperature for 5 min. After that, viable cells that had clear cytoplasm (colorless) and non-viable cells that had blue cytoplasm were counted by using a Neubauer hemocytometer. Finally, percentage of viable cells was calculated as follows: Cell Viability (%)=number of viable cells ÷ number of total cells × 100. Cell counts were expressed as mean ± standard deviation (SD).

### Colony Formation Assay (CFA)

In vitro cell survival assay based on the ability of a single cell to grow into a colony is named Clonogenic Assay or Colony Formation Assay. To evaluate the effects of *Z. multiflora* Boiss., essential oil on colony forming potential of HCT116 and SW48 cell lines, Colony Forming Assay was performed. For this, 1000 cells per well were seeded on a 6-well plate and incubated. Overall, 24 h after incubation, media were removed and cells were rinsed with PBS, then cells were treated with *Z. multiflora Boiss.,* essential oil (with concentrations of 0, 20, 40, 50, 100, 250 ppm) and incubated for 48 h. Then, the media were replaced by fresh media without drug and the cells were incubated for 18 d until cells in control plates have formed colonies. After 18 d of incubation, cultured cells were washed with PBS, thereafter, the plates were fixed with methanol: acetic acid (3:1) for 10 min, and stained with 0.5% crystal violet and left at room temperature for 30 min to dry (14). The colonies were counted using an inverted microscope (Zeiss, Axiovert 405M; Germany). Colonies, defined as 50 or more cells, were counted and plotted as a percentage of the control (untreated) colonies (15).

### Evaluation of cellular morphology

The effects of the *Z. multiflora* Boiss., essential oil on the morphology of HCT116 and SW48 colorectal cell lines were observed using phase-contrast microscopy and were photographed prior to and after treatment with IC_50_ values of this essential oil, to document possible changes in morphology.

### Acridine Orange (AO) and Ethidium Bromide (EB) Double Staining

EB/AO stain determines the membrane integrity of a cell-based on the uptake or exclusion of a dye from the cell (16). Ethidium bromide is a dye that stains only cells that have lost membrane integrity and makes cells red. Acridine orange is a membrane-permeable dye that will stain both live and dead cells and makes cells green (17). The effects of *Z. multiflora* Boiss., essential oil on HCT116 and SW48 cell lines were determined morphologically by fluorescent microscopy, after labeling with acridine orange (MERK) (Cat No. 113000) and ethidium bromide. For this purpose, HCT116 and SW48 cells were seeded in six-well plates and incubated. After 24 h, cells were treated with 0, 50, 100, 200 ppm of *Z. multiflora* Boiss., essential oil and incubated. 48 h after treatment, cell suspension was collected by centrifugation and washed with PBS and then resuspended in 10 μl of dye mixture containing 10.0 *μ*L of Acridine orange (10.0 *μ*g/mL) and 10.0 *μ*L of Ethidium bromide (10.0 *μ*g/mL) and mixed gently. Then, 10.0 *μ*L of mixed suspension was placed on a microscope slide and covered with coverslips and the morphological changes examined under fluorescence microscope.

### Measuring Apoptosis Using Flow Cytometry

Apoptosis was measured by Annexin V-FITC and propidium iodide (Annexin-V-FITC Apoptosis Detection Kit, Cat. No.: A2214-Sigma). HCT116 and SW48 cells were seeded at approximately 1 × 10^5^ cells per well in 6-well plates and allowed to adhere in 24 h at 37 °C in 5% CO_2_ incubator. Then old medium was replaced with fresh medium plus 10% FBS and supplemented with 50 ppm and 42 ppm of *Z. multiflora* Boiss. essential oil respectively for HCT116 and SW48 cells and incubated. 48 h after treatment, Cells were harvested and washed with PBS and re-suspended in 2000 *μ*L of 1X Binding Buffer then gently mixed. After that 500 *μ*L of cell suspension was transferred to four 5 ml culture tube. The first tube is cells without stain. To second and third tube, 5 *μ*L Annexin V-fluorescein isothiocyanate (*Annexin V*) was added. All the tubes incubated at room temperature for 15 min in the dark. Then to third and fourth tubes 3 *μ*L of Propidium Iodide (PI) was added. The samples were analyzed on a BD FACS Calibur flow cytometer (Becton, Dickinson and Company, Palo Alto, CA, USA). Data analysis was performed with FlowJo software (Becton, Dickinson, and Company).

### Determination of intracellular reactive oxygen species (ROS) Production

HCT116 and SW48 cells were seeded onto 6-well plates (at approximately 1 × 10^5^ cells/well) and incubated overnight. Then, cells were treated respectively with 50 ppm and 42 ppm of *Z. multiflora* Boiss. essential oil and incubated. After 48 h, Cells were harvested and washed twice with PBS. 100 μM of 1X 2′,7′-dichloro-dihydro-fluorescein diace-tate (DCFH-DA; Sigma-Aldrich, St. Louis, MO) was added to the cells and incubated for 45 min. Samples immediately were analyzed on a BD FACS Calibur flow cytometer (extinction wavelength: 485 nm and emission wavelength: 530 nm).

### Gene expression analysis by real-time quantitative PCR

3×10^3^ of HCT116 and SW48 cells were seeded in T25 flask and incubated at 37 °C in 5% CO_2_. Next day, cells were stimulated with IC_50_ concentrations of *Z. multiflora* Boiss. essential oil and incubated for 48 h. Then total RNA was isolated using RNX-Plus solution (Cat. No.: RN7713C, Cinnagen, Iran) according to manufacturer’s instructions. The quantity and quality of extracted RNA were respectively assessed by Nanodrop and by running 3μl of RNA in 1% agarose gel. Then, 500 ng of RNA from each sample was reversely transcripted by using Easy cDNA Synthesis Kit (Pars tous, Iran). Real-time PCR was performed with a Rotor-Gene Q system (Cat. No.: 9001560, Qiagen, Germany) using YTA SYBER Green qPCR Master Mix 2X (Cat. No.: YT2551, Yekta Tajhiz Azma, Iran). In a total volume of 20 in a capillary tube, 300 ng of cDNA samples, 10 μl SYBR Green Master Mix, 0.5 μl of forward and reverse primers (10 pmol), and up to 20 of nuclease-free water (Qiagen, Hilden, Germany) were added. To validate single PCR product of each primer, melting curves were analyzed. The oligonucleotide sequences and relevant product size are listed in Table 1, and *GAPDH* gene was selected as an internal reference. The primer sets were purchased from TAG Copenhagen (Copenhagen, Denmark). Relative expression was calculated using the Livac or 2 ^−ΔΔCt^.

**Table 1:** Nucleotide sequences of the primers used for real-time RT-PCR

***Gene***	***Primer***	***Seqences (5′ -> 3′)***	***Product Length***
UCP2	Forward	5′-TCAGAATGGTGCCCATCACA-3′	86 bp
	Reverse	5′-CCGGTTACAGATCCAAGGAGAA-3′	
BAX	Forward	5′-CCCGAGAGGTCTTTTTCCGAG-3′	155 bp
	Reverse	5′-CCAGCCCATGATGGTTCTGAT -3′	
BCL2	Forward	5′-CGGTGGGGTCATGTGTGTG-3′	90 bp
	Reverse	5′-CGGTTCAGGTACTCAGTCATCC-3′	
BIK	Forward	5′-ATCTTGATGGAGACCCTCCTGT-3′	134 bp
	Reverse	5′-TCACTGCCCTCCATGCATT-3′	
BAK	Forward	5′-TGGTTCTGTTGGGCCAGTTT-3′	256 bp
	Reverse	5′-CAGAGAGAGGGCACTAGCAC-3′	
CYCS	Forward	5′-TGTGCCAGCGACTAAAAAGA-3′	103 bp
	Reverse	5′-CCTCCCTTTTCAACGGTGT-3′	
GAPDH	Forward	5′-CAATGACCCCTTCATTGACC-3′,	135 bp
	Reverse	5′-TGGAAGATGGTGATGGGATT-3′	

### Statistical Analysis

For statistical analysis SPSS 22 Software (Chicago, IL, USA) was used. The data are expressed as mean ± standard deviation (SD). All the experiments in this work were performed in duplicate or triplicate. First, for the assessment of normality, we used Kolmogorov-smirnov(K-S) test. Our data are non-normal. Because they are obtained from a non-normal distribution, Kruskal–Wallis test and by Mann–Whitney test, that both are non-parametric test, were used. The statistical significance of differences throughout this study was calculated by Kruskal–Wallis test and by and *P-*value ≤0.05 was considered statistically significant.

## Results

**Fig. 1:**
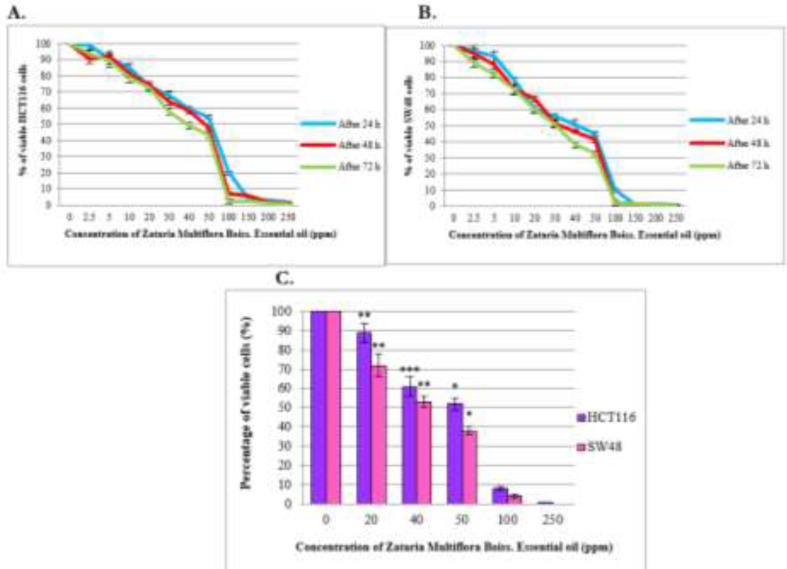
Effects of *Z. multiflora* essential oil on cell viability of HCT116 cell line (A) and SW48 cell line (B) at different concentrations at three time intervals by MTT assay. (C) Cell viability measured by trypan blue exclusion assay. Viability of HCT116 and SW48 cells 48h after treatment with various concentrations of *Zataria multiflora* Boiss. essential oil (0, 20, 40, 50, 100, 250 (ppm)) are shown. Each data is expressed as the mean ± SD obtained from three independent experiments. Statistical significance was defined at ^*^P<0.05, ^**^P<0.01 and ^***^P<0.001 Compared to control group

The results of MTT assay showed that the treatment of cells moderately reduced metabolic activity of both cell lines in dose and time-dependent manner (Fig. 1).

**Fig. 2:**
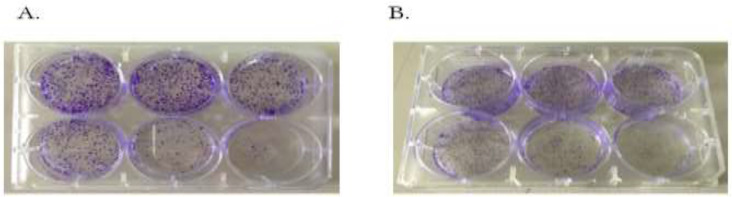
Colony formation assay of HCT116 (A) and SW48 (B) at 18th day after treatment with *Z. Multiflora* Boiss. essential oil (with concentrations of 0, 20, 40, 50, 100, 250 ppm)

The IC_50_ values of *Z. multiflora* Boiss. essential oil, that kill 50% of treated cell lines compared to untreated cells, after 24 h, 48 h and 72 h in HCT116 are 65.02, 50.93 and 44.18, and in SW48 are 48.64, 42.66 and 35.84 respectively. From results of trypan blue dye exclusion test, the viable cell count started decreasing in the treated group and this effect were observed in a concentration dependent manner (Fig. 1). The results of clonogenic assay showed that treatment with *Z. Multiflora* Boiss. essential oil significantly decreased the capacity of these tumor cells to establish colonies to a highly significant degree in a concentration dependent manner (Fig. 2). Moreover, the results of colony formation assay confirmed data of MTT and Trypan blue exclusion assays.

**Fig. 3:**
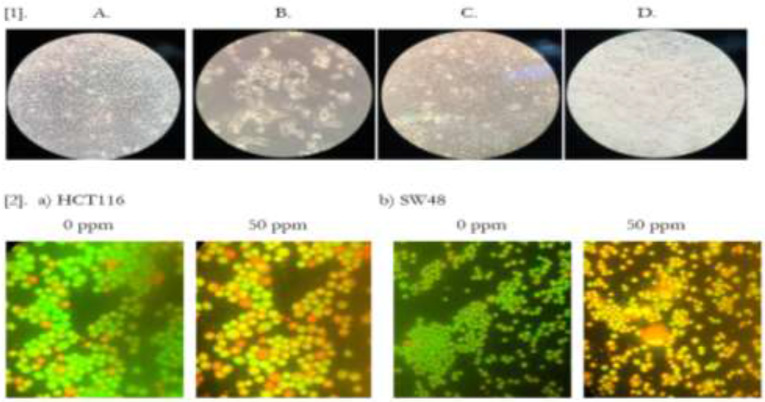
[1].Morphological characteristics of the cell lines observed under a phase-contrast microscope. (A) HCT116 cells prior to treatment and (B) HCT116 48 h after treatment with 50 ppm of *Z. Multiflora* Boiss. essential oil. (C) SW48 cells prior to treatment and (D) SW48 cells 48 h after treatment with 42 ppm of *Z. Multiflora* Boiss. essential oil. [2].Apoptotic morphological changes of HCT116 and SW48 cells detected with acridine orange/ethidium bromide staining and visualized under fluorescence microscope (a) Morphology of HCT116 cells 48 h after treatment with 0, 50 ppm of *Z. Multiflora* Boiss. essential oil, (b) Morphology of SW48 cells 48 h after treatment with 0, 50 ppm of *Z. Multiflora* Boiss. essential oil

**Fig. 4:**
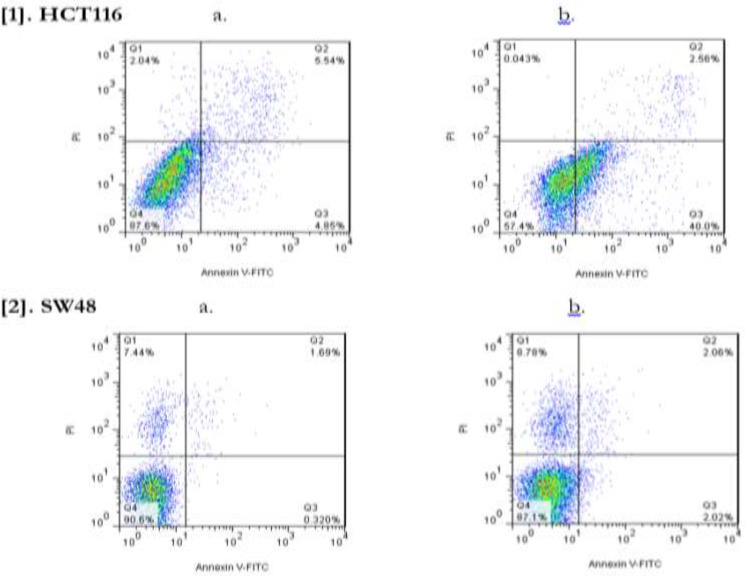
Effects of *Z. multiflora* Boiss. essential oil on apoptosis. [1] a. HCT116 cells, untreated b. HCT116 cells 48 hours after treatment with *Z. multiflora* Boiss. essential oil. [2] a. SW48 cells, untreated, b. SW48 cells, 48 hours after treatment with *Z. multiflora* Boiss. essential oil.

**Fig. 5:**
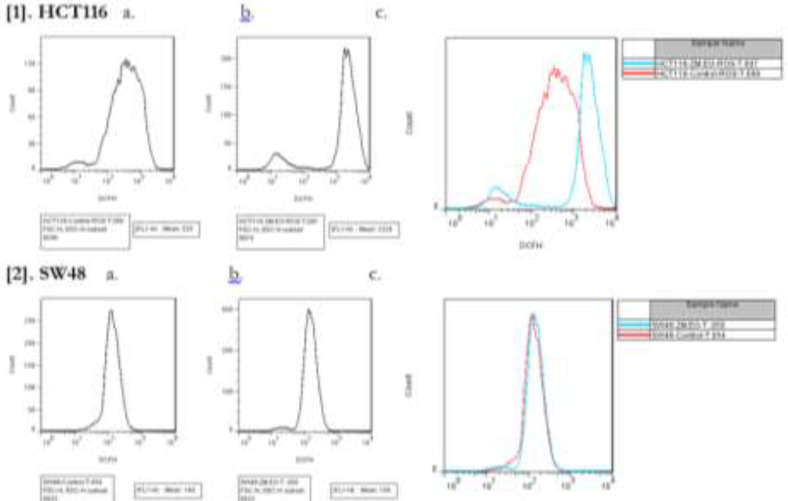
Evaluation of cellular ROS levels by DCFH-DA [1] untreated HCT116 cell line (control) (a) HCT116 cell line after treatment with *Z. multiflora* Boiss. essential oil (b) comparison between two groups (c). [2] untreated SW48 cell line (control) (a) SW48 cell line after treatment with *Z. multiflora* Boiss. essential oil (b) comparison between two groups (c)

Direct observation under a light microscope was used to assess the morphological changes of HCT116 and SW48 cell cultures after treatment with *Z. Multiflora* Boiss. essential (Fig. 3). In present study, the apoptosis, necrosis and viability of colorectal cancer cell lines treated with this essential oil were evaluated under a fluorescence microscope using acridine orange and ethidium bromide stains (Fig. 3). Cell shrinkage, membrane blebbing, chromatin condensation, and nuclear fragmentation were noticed in the HCT116 and SW48 cells after 48 h of treatment, indicating that *Z. multiflora* Boiss. essential oil induced apoptosis in these cells. In this study, *Z. multiflora* Boiss. essential oil was found to induce apoptosis in human colorectal cancer cell lines according to the results of annexin V/PI staining assay. In the cytograms, cells were untreated (up) and cells were treated with IC_50_ of *Z. multiflora* Boiss. essential oil (down) for 48 h; then, apoptosis was measured by flow cytometric analysis of cells labeled with Annexin V(x-axis) and propidiumiodide (y-axis). Representative dot plots are shown with percentages of cells displayed for each quadrant: the lower left quadrant (Q4) shows viable cells (Untreated cell), these viable cells excluded PI and were negative for Annexin V binding (annexin V- and PI-).The lower right quadrant (Q3) represents the apoptotic cells (annexin V+, PI−) demonstrating Annexin V binding and cytoplasmic membrane integrity. The upper right quadrant (Q2) represents non-viable, late apoptotic cells (annexin V+ and PI+) and the upper left quadrant (Q1) shows necrotic cells (annexin V- and PI+) (Fig. 4). Treatment of HCT116 and SW48 cells with *Z. multiflora* Boiss. essential oil for 48h, increased cellular ROS levels (Fig. 5). Totally, 48 h after incubation, average fluorescences intensity in untreated HCT116 cells and in HCT116 cells that treated with 50 ppm of *Z. multiflora* Boiss. essential oil respectively were 525 and 2224, and in untreated SW48 cells and in SW48 cells that treated with 42 ppm of *Z. multiflora* Boiss. essential oil respectively was 148 and 159. Expression of Ucp2 in HCT116 and SW48 relative to normal human fibroblast cell lines was significantly increased, and expression of Ucp2 after treatment with *Z. multiflora* Boiss. essential oil was significantly decreased. After treatment, expression of anti-apoptotic gene *Bcl2* was significantly decreased and the expression of proapoptotic effectors genes *Bax*, *Bak* and proapoptotic activators gene *Bik* was significantly increased in HCT116 and SW48 cell lines.

## Discussion

This investigation showed that *Z. multiflora* Boiss. essential oil had anticancer activity against both HCT116 and SW48 cells and induced apoptosis **via** the UCP_2_-mediated mitochondrial pathway. After treatment of HCT116 and SW48 cells with *Z. multiflora* Boiss. essential oil, the expression of *Ucp2* was downregulated. Following downregulation of *Ucp2*, the expression of *Bcl*_*2*_ was downregulated and the expression of *Bax, Bak*, *Bik*, *Cytochrome c* was upregulated in human colorectal HCT116 and SW48.

Among plant derived compounds, essential oils (EOs) are volatile compounds produced by plants that present different biological activities including antitumor effect. In the last two decades, the anticancer potential of Essential oils and their components were explored in vitro and in vivo models (2). The essential oil of *Z. Multiflora* Boiss., was acquired by hydrodistillation and decomposed it by Gas Chromatography and Gas Chromatography - Mass Spectrometry. It was discovered 48 ingredients in this essential oil as the main ingredients were Carvacrol (More than half of it), Linalool, p-Cymene, Thymol (18). *Z. Multiflora* from Shiraz Province of Iran was discovered the main ingredients of *Z. multiflora* Boiss. essential oil was Carvacrol (71.1%) (5). Moreover, carvacrol have strong antitumor activity. For example, the anti-tumor effects of carvacrol was demonstrated on human metastatic breast cancer cells, MDA-MB 231 (2). The mRNA expression level of UCP_2_ gene was assessed in colon cancer and they found that *UCP*_*2*_ gene was upregulated in colon cancer tissue samples than in its adjacent tissue samples. They also revealed that the high UCP_2_ expression level was significantly correlated to colon cancer metastasis (19). Cytotoxic effects of Hydro-alcoholic extract of *Z. multiflora* was reported on HT-29 and SW-48 colon cancer cell lines using MTT assay (20).

## Conclusion

*Z. multiflora* Boiss. essential oil was able to significantly inhibit cell proliferation, colony formation, and induce cell apoptosis and induced cytotoxicity in cultured colon cancer cell lines, as well as suppress tumor growth in vivo. The *Z. multiflora* Boiss. essential oil is a promising drug candidate for the treatment of colon cancer.

## Ethical considerations

Ethical issues (Including plagiarism, informed consent, misconduct, data fabrication and/or falsification, double publication and/or submission, redundancy, etc.) have been completely observed by the authors.
